# Unveiling human origins of replication using deep learning: accurate prediction and comprehensive analysis

**DOI:** 10.1093/bib/bbad432

**Published:** 2023-11-25

**Authors:** Zhen-Ning Yin, Fei-Liao Lai, Feng Gao

**Affiliations:** Department of Physics, School of Science, Tianjin University, Tianjin 300072, China; Department of Physics, School of Science, Tianjin University, Tianjin 300072, China; Department of Physics, School of Science, Tianjin University, Tianjin 300072, China; Frontiers Science Center for Synthetic Biology and Key Laboratory of Systems Bioengineering (Ministry of Education), Tianjin University, Tianjin 300072, China; SynBio Research Platform, Collaborative Innovation Center of Chemical Science and Engineering (Tianjin), Tianjin 300072, China

**Keywords:** human genome, origin of replication, deep learning, Z-curve method

## Abstract

Accurate identification of replication origins (ORIs) is crucial for a comprehensive investigation into the progression of human cell growth and cancer therapy. Here, we proposed a computational approach Ori-FinderH, which can efficiently and precisely predict the human ORIs of various lengths by combining the Z-curve method with deep learning approach. Compared with existing methods, Ori-FinderH exhibits superior performance, achieving an area under the receiver operating characteristic curve (AUC) of 0.9616 for K562 cell line in 10-fold cross-validation. In addition, we also established a cross-cell-line predictive model, which yielded a further improved AUC of 0.9706. The model was subsequently employed as a fitness function to support genetic algorithm for generating artificial ORIs. Sequence analysis through iORI-Euk revealed that a vast majority of the created sequences, specifically 98% or more, incorporate at least one ORI for three cell lines (Hela, MCF7 and K562). This innovative approach could provide more efficient, accurate and comprehensive information for experimental investigation, thereby further advancing the development of this field.

## INTRODUCTION

During every cell cycle, the genetic information must be completely replicated, initiating at specific sites known as origins of replication (ORIs) [1, 2]. Unlike most prokaryotes with only one ORI [3–5], eukaryotes have multiple ORIs [6], which ensure the efficient and complete replication of the genetic material in large genomes [7, 8]. Previous reports indicate that the activation of ORIs is a dynamic process, making it difficult to detect all ORIs through experimental means [9]. Computational prediction of ORIs based on existing experimental data can guide experiments and alleviate the experimental workload. By identifying the potential regulatory elements and binding sites for replication factors, how ORI activation is controlled can be better understood [7]. It provides assistance for a comprehensive understanding of the eukaryotic gene structure, carcinogenesis research and therapeutic strategies [9, 10].

However, because of the limited availability of ORIs, most of existing computational methods heavily rely on traditional machine learning (ML) methods. These methodologies require the truncation of DNA sequences of varying lengths to a uniform length. For example, iORI-Euk [11] is a prediction software tool based on support vector machine, which utilized a data set comprising 5506 ORIs (2332 for K562, 2763 for MCF7 and 411 for Hela) with a length of 300 bp. These sequences are truncated before being fed into this software to predict ORIs in *Homo sapiens*. Another approach, called Stack-ORI [12], employs extreme gradient boosting (XGBoost) and the same data set as iORI-Euk [11] for training and testing. Despite the progress made by current computational methods, the truncation of ORIs may lead to the loss of replication firing function, significantly impacting the accuracy (ACC) of prediction methods. As mentioned above, two primary challenges still persist: the scarcity of available ORI data, as previous methods were mostly based on small samples, and the truncation of DNA sequences.

The advent of next-generation sequencing technologies has enabled the identification of numerous novel ORIs [13]. Nevertheless, substantial disparities exist in the results of ORIs obtained through diverse experimental methods and cell lines. To facilitate a comprehensive analysis of ORI data, several related databases have been established such as DeOri, the database of eukaryotic DNA replication origins [14] and OriDB, the DNA replication origin database [15]. These databases serve as valuable resources for studying DNA replication. Building upon the foundation of these large-scale ORI data [14], Dao *et al.* [16] incorporated epigenetic features into ORI prediction. This approach enables the prediction of ORIs of varying lengths and has exhibited highly favorable outcomes, with an AUC of 0.9627. Although this method has proven effective, it does face limitations associated with epigenetic data. Therefore, there is a recognized demand for a DNA sequence-driven prediction method.

To address these limitations, we developed Ori-FinderH, a new computational approach that combines the Z-curve method [17] and deep learning approach [18] to predict ORIs of different lengths. Through the Z-curve method, it is possible to represent DNA sequences with various lengths within a consistent-dimensional space (Z-space). By choosing diverse Z-parameters, different dimensional Z-space can be spanned. We evaluated the classification performance using the identical model in Z-space of different dimensions (12, 48, 60, 192, 204, 240 and 252). The results demonstrated that optimal performance was reached in 252-dimension Z-space (AUC = 0.9706). Moreover, we observed that after encoding with the Z-curve method, the ORIs from different cell lines were concentrated in a certain region of the Z-space, which was the high-dimensional space of Z-parameters. Accordingly, we proposed a cross-cell-line model to distinguish ORIs between different cell lines.

The *de novo* design of genomic elements plays a crucial role in enhancing our understanding of internal regulatory mechanisms, metabolic pathways and the fundamental principles governing gene expression within organisms [19–22]. Through precise manipulation and adjustment of genomic components, we can uncover the intricacies and inherent patterns of biological processes. For instance, Wang *et al.* [19] utilized generative adversarial networks to create artificial promoters for *Escherichia coli*, and Kotopka *et al.* [22] successfully designed yeast promoters using deep learning techniques. In present study, we combined a genetic algorithm (GA) [23] with our cross-cell-line model to make ORIs evolve, artificially.

## MATERIALS AND METHODS

### Benchmark data set

Prior prediction methods have frequently employed the practice of truncating DNA sequences with varying lengths to a uniform length [11, 12, 24, 25]. However, empirical evidence clearly indicates that ORIs exhibit diversity in their lengths [1, 13, 14, 26, 27], and truncation can result in the loss of their replication function. Recognizing this limitation, researchers have taken a keen interest in addressing this issue. Notably, Dao *et al.* [16] established a multicellular data set comprising a substantial number of human ORIs with varying lengths. Our study employed this data set as benchmark data set, which is available at https://github.com/linDing-group/iORI-Epi.

### Z-curve method

The Z-curve is an intuitive method for sequence visualization that can display purine versus pyrimidine, amino versus keto and strong H-bonded versus weak H-bonded bases along the DNA sequence [17, 28–30]. Sequence characteristics, including base composition distribution and periodicity patterns, are well displayed using the Z-curve method. Based on this method, numerous valuable tools for sequence analysis have been developed, contributing to our understanding of DNA sequences, and uncovering new biological insights. In general, the Z-curve method opens a new area of genome analysis using a geometric approach and provides an example of an innovative and systematic study [17, 28, 29]. For mononucleotide, ignoring the phase factor, we have:


(1) \begin{equation*} \left\{\begin{array}{@{}c}x=\left(A+G\right)-\left(C+T\right)\\ {}y=\left(A+C\right)-\left(G+T\right)\\ {}z=\left(A+T\right)-\left(G+C\right)\end{array}\right. \end{equation*}

Based on Equation (1), any given DNA sequence can be encoded into a 3D vector. In the following steps, the phase aspect will be included. The phase-specific DNA sequence is obtained based on the following equation.


(2) \begin{equation*} \left\{\begin{array}{@{}c}{S}_0={N}_1{N}_2{N}_3\dots \\ {}{S}_i={N}_i{N}_{i+3}{N}_{i+2\times 3}\dots \end{array}\right.\ i=1,2,3 \end{equation*}

where *N* can be *A*, *C*, *G* and *T*. ${S}_0$ represents the initial DNA sequence. ${S}_i$ represents the DNA sequence corresponding to phase $i$.


(3) \begin{equation*} \left\{\begin{array}{@{}c}{x}_i=\left({A}_i+{G}_i\right)-\left({C}_i+{T}_i\right)\\ {}{y}_i=\left({A}_i+{C}_i\right)-\left({G}_i+{T}_i\right)\\ {}{z}_i=\left({A}_i+{T}_i\right)-\left({G}_i+{C}_i\right)\end{array}\right.\ i=1,2,3 \end{equation*}

Once the phase-specific DNA sequence is extracted, regarding every phase-specific DNA sequence (${S}_i$), we can get a group phase-specific ${x}_i$, ${y}_i$, ${z}_i$ as shown in Equation (3). Finally, a 9D ($3\times 3$) vector is acquired.

Expanding the scope from mononucleotide, dinucleotide and trinucleotide of Z-parameters are developed. The basic principle is consistent with mononucleotide, except for the modification of Equations (1 and 3) by replacing mononucleotide with dinucleotide or trinucleotide. Here, we explored the optimal combination of Z-parameters, and the description of the Z-space in 12, 48, 60, 192, 204, 240, 252-dimensions is presented in Supplementary Table 1.

### Model architecture

**Figure 1:**
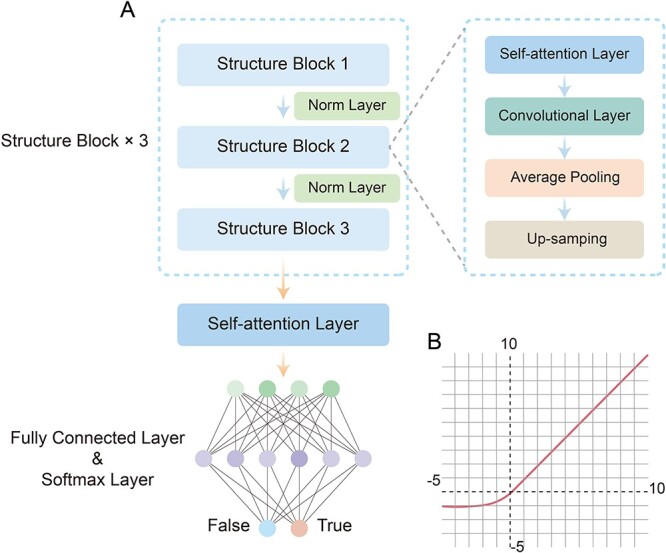
The flowchart of Ori-FinderH. (**A**) Schematic diagram of Ori-FinderH model. The entire model is primarily composed of three structure blocks, each containing a self-attention layer, convolutional layer, average pooling layer and up-sampling layer. Here, binary output is obtained via an MLP. (**B**) ELU function diagram. ELU function has negative values; therefore, the average activation value is close to 0, which makes learning faster with gradients closer to natural ones.

As illustrated in Figure 1A, the architecture of ORI-FinderH primarily consists of two components: the stacked structure block and the classification module. Each structure block encompasses a self-attention layer, which aims to capture correlations among different positions present within the output of the preceding layer [31]. Main function of self-attention is shown in Formula (4), where ${d}_k$ represents the dimensionality of the input data. The queries and keys have a dimensionality of ${d}_k$ dimension, and the values have a dimensionality of ${d}_v$ dimension. *Q*, *K* and *V* are the group of them, and the attention can be calculated as follows:


(4) \begin{equation*} \text{Attention}\left(Q,K,V\right)=\text{softmax}\left(\frac{Q{K}^T}{\sqrt{d_k}}\right)V \end{equation*}

Subsequently, a 1D convolutional layer is used to process the data from self-attention layers. After convolutional layer, average pooling is added to this model that can be employed to reduce the dimensionality of input features and extract more abstract feature representations. Toward the end of structure block, to further enhance the model’s generalization capacity and robustness, an up-sampling layer is added. The exponential linear unit (ELU) (Figure 1B) is chosen to serve as the activation function in our model.


(5) \begin{equation*} \text{ELU}(x)=\left\{\begin{array}{@{}c}x,x>0\\ {}\alpha \left({e}^x-1\right),x\le 0\end{array}\right. \end{equation*}

where $\alpha$ is a hyperparameter with the default value $\alpha =1.0$. Ultimately, by composed three structure blocks and a multilayer perceptron (MLP) our model has been constructed.

### Training and implementation

All experiments are conducted in a server with four Nvidia RTX 3090. Binary cross-entropy loss (BCE Loss) is used in train process.


(6) \begin{equation*} \text{BCE}\ \text{Loss}=-\left[y\log \left(\hat{y}\right)+\left(1-y\right)\log \left(1-\hat{y}\right)\right] \end{equation*}

The optimization process utilizes the Adam optimizer, a widely used stochastic optimization algorithm that combines the advantages of the adaptive learning rate methods and momentum-based optimization techniques. The additional hyperparameters are listed below:


(7) \begin{equation*} \left\{\begin{array}{@{}c}\text{Learning}\ \text{rate}=0.0002\\ {}\text{Batch}\ \text{size}=64\end{array}\right. \end{equation*}

K-fold cross-validation is a commonly used technique in scientific research, particularly for data analysis and ML. On account of the same benchmark data set, proposed method can be fairly compared with iORI-Epi [16], which is the state-of-the-art method for predict ORIs with various length. For objective comparison, both methods undergo 10-fold cross-validation, that is, the data set is divided into 10 parts, with nine parts used for training and one part used for model evaluation in each iteration. During practical implementation, Sklearn is used to split data set.

### GA framework

**Figure 2:**
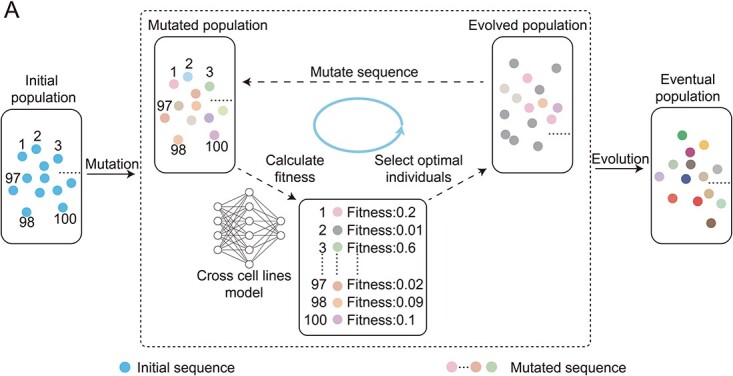
The flowchart of genetic algorithm. Each DNA sequence is denoted by an individual dot.

GA is a collection of algorithms that mimics biological evolution [32]. In this study, we integrate a deep learning model as the fitness function into the GA framework (Figure 2). The integration allows us to simulate and generate a set of human ORIs. To initiate this process, we create an initial population derived from sequences, each comprising 1000 base pairs and exclusively containing adenine residues (A) or guanine residues (G).

Next, we proceed with mutating each sequence within the initial set. Four distinct mutation modes are designed: point mutation, insertion mutation, deletion mutation and structural variation. After mutation, we employ deep learning to quantify the fitness of each sequence. Sequences exhibiting higher fitness levels are selected for inheritance by the subsequent generation. Through iterative refinement, the algorithm terminates when it attains the optimal fitness level (where the minimum fitness exceeds 0.5) for the sequence, at which point the final outcome is generated.

### Bioinformatics software

Multiple EM for Motif Elicitation (MEME) software [33] is performed with default parameters to identify the motifs in ORI sequences. CD-HIT [34] is used to remove sequences with more than 80% pairwise sequence identity to any other in the benchmark data set in order to eliminate redundancy and prevent bias.

## RESULT

### The ORIs in human genome can be predicted with high performance by combination of Z-curve method and deep learning

DNA replication in multicellular organisms is a dynamic and plastic process, and errors in its initiation can result in many diseases, including Meier–Gorlin syndrome, X-linked pigmentary reticulate disorder and cancer [9, 10, 35]. Particularly, ORIs contain essential components for the initiation of DNA replication.

Therefore, there is an urgent need to establish a computational model that can rapidly predict the locations of ORIs. Nonetheless, most existing methods truncate ORI sequences into the same length for ML analysis, which would result in the obtained ORIs possibly losing functionality. In contrast, our method facilitates computational processing while preserving the inherent crucial features of the original ORI sequences.

**Figure 3:**
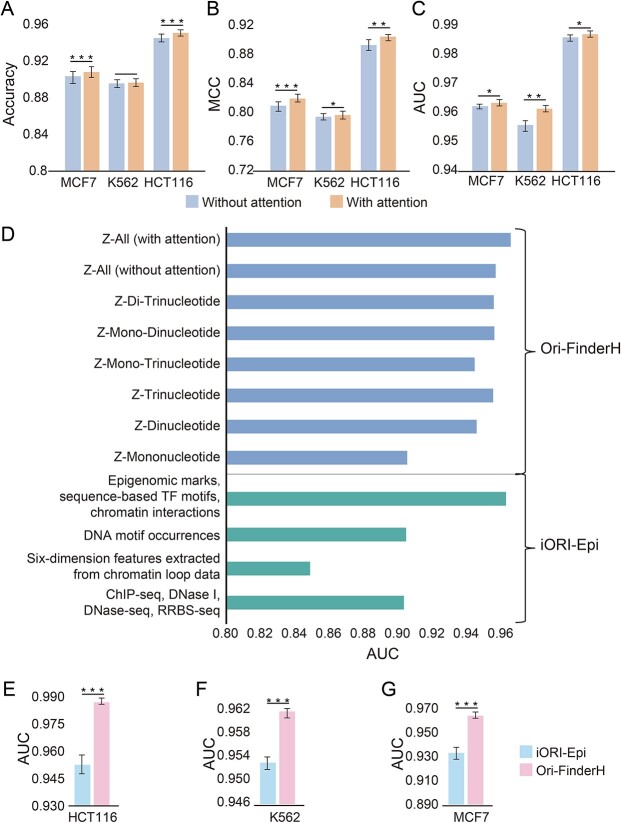
Comprehensive performance of the present model. (**A**–**C**) Cross-cell-line performance comparison between with and without attention for ACC (A), MCC (B) and AUC (C), respectively. (**D**) Comparison of different parameters and methods. (**E**–**G**) Cross-cell-line performance comparison between iORI-Epi and Ori-FinderH in HCT116 (E), K562 (F), MCF7 (G), respectively. Note: ^*^ means *P* < 0.05, ^*^^*^ means *P* < 0.01, ^*^^*^^*^ means *P* < 0.001.

Novel methods such as iORI-Epi [16] identify ORIs based on epigenetics. In this study, Z-curve encoding was applied to process original ORI sequences of varying lengths, thereby transforming them into uniform-length arrays. The prediction performances of the encoding methods with Z-curve parameters of mononucleotide, dinucleotide and trinucleotide were evaluated using the same benchmark data set (Figure 3D). Among these, the encoding method using the Z-curve parameters of trinucleotide (AUC = 0.9552) demonstrated superior predictive performance. Furthermore, when employing hybrid Z-curve parameters, the combination of mononucleotide, dinucleotide and trinucleotide exhibited the highest performance (AUC = 0.9567), and its performance was closest to that of the mixed epigenetic method of iORI-Epi (AUC = 0.9627). Consequently, our approach demonstrated remarkable efficacy in accurately predicting human ORIs.

To further enhance the model’s performance, a self-attention layer [31] was incorporated into the original model. With the integration of self-attention, the test performance of the model exhibited a significant improvement across all three cell lines (Figure 3A–C). The performance was evaluated using three metrics, namely, ACC, Matthew’s correlation coefficient (MCC) and AUC. Specifically, the AUC increased from 0.9862 to 0.9872 for HCT116 cells, from 0.9559 to 0.9616 for K562 cells and from 0.9624 to 0.9636 for MCF7 cells. Using the same data set [16], a comparative analysis was conducted between the proposed model and existing iORI-Epi [16] method. Our model outperformed the previous method in identifying ORI sequences across the three distinct cell lines (Figure 3E–G). For HCT116 cells, an AUC of 0.9527 was demonstrated using iORI-Epi, whereas our model exhibited a substantially improved AUC of 0.9872. Similarly, for MCF7, an AUC of 0.9329 was obtained by iORI-Epi, whereas an increased AUC of 0.9636 was obtained by our model. Furthermore, for K562, an AUC of 0.9532 was obtained by iORI-Epi, whereas an AUC of 0.9616 was manifested by our outcome.

### The average GC content of ORIs is notably higher than that observed in the human genome, and there are numerous GC-rich motifs in ORIs

**Figure 4:**
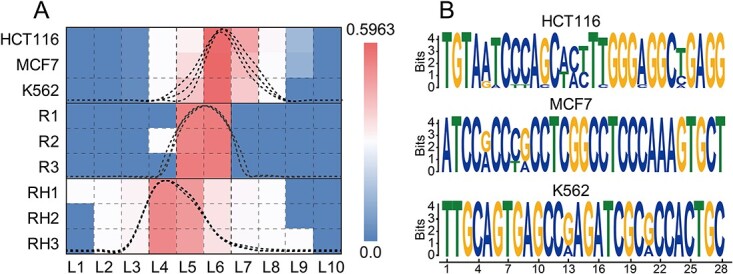
Statistical analysis of GC contents and GC-rich motifs in ORIs. (**A**) Statistical diagram of GC content distribution for different sequence sets. Li represents GC content between 10(i–1)% and 10i% (1 ≤ i ≤ 10). (**B**) The GC-rich motifs discovered in the ORIs from different cell lines.

The distribution of nucleotide in the genome is uneven, with higher GC or AT content in certain regions that potentially contain important genomic elements [36, 37]. To investigate this uneven distribution of ORIs, sequence sets were randomly generated using computer (R1–R3) or were intercepted from the human genome (RH1–RH3) for comparison. The GC content distributions were then calculated between them and the ORI sequences (Figure 4A). It was observed that the GC content of sequences randomly generated by computer was primarily ~40–60% because of the equal probability of nucleotide occurring in these sequences, whereas the GC content of randomly intercepted sequences from the human genome primarily ranged between 30 and 50%. However, the GC content of the ORIs primarily ranged between 50 and 60%, which was significantly higher than those in the above sequence sets.

**Figure 5:**
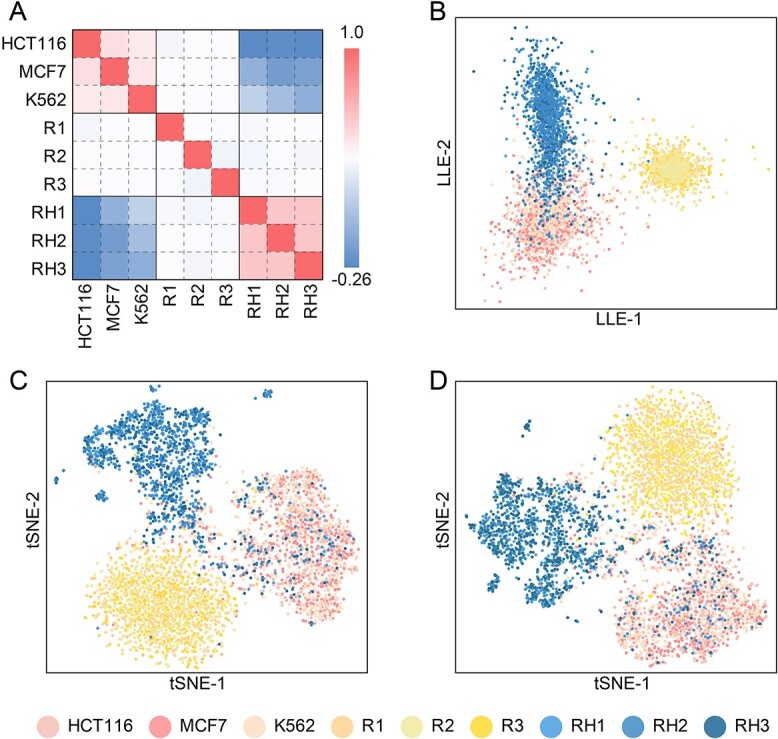
The cosine distance and dimensionality reduction analysis between different sequences after Z-curve encoding. (**A**) The cosine distance between different sequences after Z-curve encoding. (**B**) The result of dimensionality reduction analysis using LLE on the sequences after Z-curve encoding. (**C**, **D**) The result of dimensionality reduction analysis using t-SNE on the sequences after Z-curve encoding.

To better identify genomic elements in ORI sequences, potential motifs within ORI sequences were identified using the MEME software [33], which revealed a substantial number of motifs with high GC content (Figure 4B). Further analysis based on transcription factor data from the Jaspar database [38] indicated that most of these GC-rich motifs correspond to Paired-related HD factors [39] and C2H2 zinc finger factors [40], which are known to be growth- and development-related transcription factors. The present analysis is consistent with the findings of previous studies [16].

### ORI sequences have a propensity to form a concentrated cluster within a distinct region of Z-space

To explore the distributions of the above sequence sets in Z-space, they were encoded using the Z-curve method and mapped together into Z-space. To ascertain the feasibility of isolating these ORIs from the other two types in the high-dimensional Z-space, we computed the mean cosine distance among the ORIs (HCT116, MCF7 and K562), randomly generated sequences by computer (R1–R3) and human genome-based sequences (RH1–RH3).

**Figure 6:**
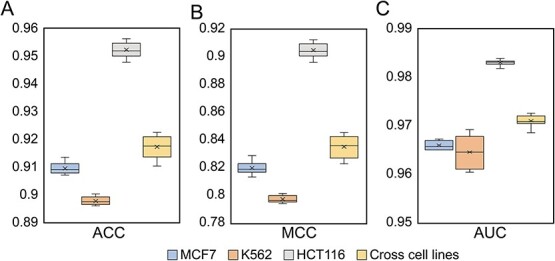
The performance of 10-fold cross-validation test between models established for different cell lines and cross-cell-line models.

**Figure 7:**
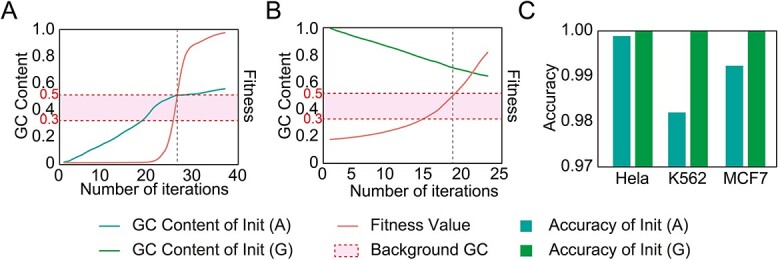
Analytical results of generated ORIs. (**A**) The GC content and fitness for generated ORIs from the initial sequences containing only A, which is abbreviated to Init (A). (**B**) The GC content and fitness for generated ORIs from the initial sequences containing only G, which is abbreviated to Init (G). (**C**) The test results obtained by using iORI-Euk to discriminate the generated ORIs.

The cosine distance is a widely used metric in diverse scientific fields to evaluate vector similarity based on the cosine value of the angle between them. As the cosine distance tends toward 1, this indicates a stronger resemblance between vectors, whereas values approaching 0 imply a reduced similarity or lack of correlation. Conversely, a value nearing −1 indicates an opposition between vectors [41]. Our analysis revealed a discernible degree of sequence similarity between ORIs from diverse cell lines (Figure 5A). In contrast, the randomly generated sequences (R1–R3) exhibit no apparent association with either of the other two sequence sets, with cosine distance values approaching 0. Some associations are observable between the human genome-based sequences and ORI sequences. A negative cosine distance value suggests that the ORI sequences and human genome-generated sequences (RH1–RH3) are opposites. Building on these findings, locally linear embedding (LLE) [42] and t-distributed stochastic neighbor embedding (t-SNE) [43] were employed to project the encoded sequences onto a 2D plane, which delineated their clustering patterns more accurately.

### Based on the Z-curve encoding approach, it is feasible to develop a cross-cell-line model for predicting human ORIs

Our findings indicate that encoded ORI sequences display a clustering pattern in space compared with non-ORI sequences. In fact, an attempt was made to further classify ORIs and initially distinguish them according to their cell lines. We developed a multi-classification model using the same architecture as depicted in Figure 2A, for K562, MCF7 and HCT116 cells. However, even after multiple rounds of training, the ACC remains around 0.5, MCC is ~0.1 and AUC is ~0.5. These results indicate that the precise classification of encoded ORIs according to their respective cell lines is not possible. Therefore, it can be inferred that the ORIs encoded by the Z-curve method display a consistent pattern, which provides a potential avenue for establishing a cross-cell-line prediction model for human ORIs. To validate this hypothesis, the model was trained based on amalgamated ORI data from the three cell lines. The results of the 10-fold cross-validation test are depicted in Figure 6. In contrast to models tailored to individual cell lines, the cross-cell-line model exhibited superior recognition efficacy in the MCF7 and K562 cell lines. The recognition capability decreased in the HCT116 cell line. Nevertheless, a comprehensive evaluation of all test metrics yielded an excellent performance (ACC = 0.9172, MCC = 0.8352, AUC = 0.9706).

### The artificial generation of ORIs using GA and deep learning is feasible

In the generation of artificial ORIs, a trained cross-cell-line model is employed as the fitness function in a GA. The initial set of sequences exclusively consisted of DNA sequences containing adenine (A), resulting in a GC content of 0. However, after multiple evolutions, the GC content ultimately stabilized at 50–60% (Figure 7A). Subsequently, we transitioned to an initial set of sequences exclusively containing guanine (G). As the GC content decreased to 60%, we observed a significant increase in the fitness function (Figure 7B). This GC content matched the previous analysis of the ORIs (Figure 6A). The generated ORIs were presented in Supplementary Table 2.

Furthermore, we evaluated the generated ORIs using iORI-Euk [11] (Figure 7C). For initial sequences with only adenine, 98% of them contained at least one ORI. Similarly, for initial sequences composed solely of guanine, each sequence contained at least one ORI. The detailed test results can also be found in Supplementary Table 2. In conclusion, our results suggest the feasibility of employing a deep learning model as the fitness function for a GA to generate ORIs.

## CONCLUSION

In this study, a novel model for human ORI prediction was developed using Z-curve method and deep learning approach. By incorporating the self-attention mechanism, the proposed model demonstrated a remarkable performance in predicting human ORIs of different lengths. The accurate prediction of ORI locations could have significant implications on the study of DNA replication and its molecular mechanisms. By providing a reliable predictive model for human ORIs, this study has the potential to facilitate the design and execution of subsequent experiments, thereby reducing the workload required for experimental verification. The inclusion of the self-attention mechanism in the proposed model is particularly noteworthy, as it allows the model to effectively identify and focus on relevant features within the input data, thereby improving its performance in ORI prediction. To further explore the characteristics of ORIs, we employed GA and deep learning approach to establish a process for artificially generating ORIs. A third-party ORI prediction software tool was employed to assess the artificial ORIs, yielding highly favorable results. Future research would extend the explanations of ORI features, and subsequent experiments can be conducted to validate the activity of the artificial ORIs *in vivo*.

**Key Points**

- The human origin of replication (ORI) sequences were encoded and accurately recognized by using the Z-curve method and deep learning fusion.
- The present method can deal with the human ORIs of various lengths and outperformed the state-of-the-art prediction method.
- The method of artificially generating ORI sequences was proposed based on the present deep learning model.

## Supplementary Material

supplementary_table_1_bbad432

supplementary_table_2_bbad432

## Data Availability

The benchmark data sets used in this study are freely available at https://github.com/linDing-group/iORI-Epi.

## CODE AVAILABILITY

All codes are freely available in our GitHub repository (https://github.com/tubic/Ori-FinderH).

## Author Biographies

**Zhen-Ning Yin** is a PhD student in the Department of Physics, School of Science, Tianjin University. His research interests are computational biology, deep learning and bioinformatics.

**Fei-Liao Lai** is a PhD student in the Department of Physics, School of Science, Tianjin University. His research interests are computational biology, deep learning and bioinformatics.

**Feng Gao** is a professor in the Department of Physics, School of Science, and the Frontiers Science Center for Synthetic Biology and Key Laboratory of Systems Bioengineering (Ministry of Education), Tianjin University. His researches are performed in the fields of computational biology and bioinformatics.
